# Different Roles of Rumination and Mindfulness among Cyber-Ostracized Adolescents’ Psychological Well-Being

**DOI:** 10.3390/ijerph19031222

**Published:** 2022-01-22

**Authors:** Xue Li, Wenlong Mu, Yu Wang, Peng Xie, Yuwei Zhang, Ting Liu

**Affiliations:** 1 School of Foreign Studies, Zhongnan University of Economics and Law, Wuhan 430073, China; xueli@zuel.edu.cn; 2 School of Economics and Management, Wuhan University, Bayi Road, Wuchang District, Wuhan 430072, China; mu.wenlong@outlook.com (W.M.); pengxie@whu.edu.cn (P.X.); zhangyuw@whu.edu.cn (Y.Z.); lexi9899@whu.edu.cn (T.L.)

**Keywords:** cyber-ostracism, rumination, mindfulness, psychological well-being, adolescents

## Abstract

Previous research has confirmed the harmful effects of cyber-ostracism on adolescents. However, research that has investigated the effect of cyber-ostracism on adolescents’ psychological well-being and the underlying mechanisms of this influence remains scarce. Using a sample of 421 Chinese adolescents, this study examined the short-term effect of cyber-ostracism on adolescents’ psychological well-being, along with the mediating effect of rumination. Mindfulness is considered as a moderator influencing this underlying mechanism. Questionnaires regarding cyber-ostracism, rumination, and mindfulness were administered at the beginning of the spring semester. Psychological well-being was assessed three months later. The study found that cyber-ostracism significantly and negatively predicted adolescents’ psychological well-being. As shown by the mediation analysis, rumination partly mediated the effect of cyber-ostracism on adolescents’ psychological well-being. Moderated mediation analysis indicated that mindfulness played a moderating role in the relationship between cyber-ostracism and adolescents’ psychological well-being as well as the relationship between cyber-ostracism and rumination. Specifically, mindfulness would decrease the negative impact of cyber-ostracism on adolescents’ psychological well-being. This study uncovers the short-term effect of cyber-ostracism on adolescents’ psychological well-being and accentuates the underlying mechanisms of this effect, which has substantial implications for interventions and practices to reduce the detrimental effects of cyber-ostracism among adolescents.

## 1. Introduction

Social networking sites (SNSs) have gradually replaced some “face-to-face” communication for adolescents and have rapidly become an indispensable social tool for connection [1,2]. Social interaction through SNSs enables adolescents to establish and maintain relationships [3,4], develop social identity [5], and obtain acceptance and approval from others [6]. For instance, a study conducted by Beyens et al. [7] unveiled that the motivation to establish and sustain positive and steady interpersonal connections was positively linked to higher usage of Facebook in adolescents. However, not everyone can successfully build up social connections and obtain acceptance on SNSs. Once an individual cannot fulfill the abovementioned needs, they will perceive the sentiments of being ignored and excluded, which is known as cyber-ostracism [8].

Cyber-ostracism is not just simply an alternative form of in-person ostracism experienced in an online environment [9,10]; there are several specific traits that characterize cyber-ostracism as different from in-person ostracism. First, due to the asynchronism of online communication, cyber-ostracism is more likely to be elicited than in-person ostracism [11]. For example, waiting for “likes” and “comments” after posting a status update on Facebook can be stressful and easily trigger the perception of cyber-ostracism [12,13]. Cyber-ostracism can also be experienced when there is a lack of response from sent messages such as emails or texts [14]. In another study, Büttner and Rudert [15] found that not being tagged in a posted photo on social media platforms could elicit the feeling of cyber-ostracism. Another characteristic of cyber-ostracism is the ambiguity or uncertainty it presents due to the reason that cyber-ostracized individuals are often unsure and confused on why it is occurring [16]. Moreover, cyber-ostracism is more public and persistent than offline ostracism because the absence of feedback is often visible for others to see and continuously retained on SNSs.

Compared to other age groups, adolescents are a group of people who are particularly sensitive and susceptible to cyber-ostracism, as they are undergoing a phase where they long for social acceptance and affiliation by peers [17,18]. The extant literature has confirmed the detrimental effects of cyber-ostracism on adolescents’ mental health, such as emotional well-being [10] and depression [16], yet research that investigated the effect of cyber-ostracism on adolescents’ psychological well-being remains scarce The psychological well-being of adolescents is associated with healthy behaviors [19], positive development [20], and a lower risk for aggressive behavior and psychological disorders [21]. Therefore, it is necessary to investigate the association between cyber-ostracism and the psychological well-being of adolescents, along with the mechanisms underlying this association. The findings can advance our understanding of adolescents’ cyber-ostracism as well as enrich the existing theoretical literature on well-being and ostracism. The implications can shed light on interventions that aim to alleviate the detrimental effects of cyber-ostracism on adolescents’ psychological well-being.

### 1.1. Cyber-Ostracism and Psychological Well-Being

Well-being is often studied from two distinct dimensions: hedonic and eudaimonic approaches [22,23,24]. Hedonic well-being, also referred to as emotional well-being, is defined as a positive emotional state that an individual experiences at the moment [24,25] Eudaimonic well-being, also known as psychological well-being, describes well-being as a state of optimal human functioning (e.g., self-actualization, personal growth, and pursuit of meaning in life) that extends beyond the experience of pleasure, also entails the fulfillment of one’s real potential [24,26]. Emotional well-being and psychological well-being are distinct constructs [27] that differ in their level of stability [28] and their association with other constructs [29]. Emotional well-being is relatively unstable and highly susceptible to stressful life events [30]. However, psychological well-being is a relatively stable construct that is not easily affected by short external stimulation [28].

So far, several empirical studies have explored the impact of cyber-ostracism on both emotional and psychological well-being. For instance, using an experimental design, Smith, Morgan, and Monks [10] found that cyber-ostracized individuals reported lower levels of emotional well-being than cyber-included individuals. In another study, Schneider et al. [9] adopted a new experiment paradigm to manipulate cyber-ostracism and found that the stimulation significantly undermined emotional well-being but did not have a significant influence on psychological well-being. Wang et al. [31] replicated Schneider et al.’s study using a Chinese sample and found that cyber-ostracism simultaneously weakened both emotional and psychological well-being. However, the effect size of the association between cyber-ostracism and psychological well-being was relatively small (*f* = 0.22). We propose that the inconsistent findings regarding the relationship between cyber-ostracism and psychological well-being are attributed to two reasons. First, prior research often used the experiment paradigms to investigate the effect of cyber-ostracism experience on psychological well-being [16]. However, due to the relatively stable feature of the psychological well-being construct [28], it is not easily affected by the brief stimulation of cyber-ostracism [9,31]. It is important to focus on the general situation of cyber-ostracism, which captures the extent to which individuals experience cyber-ostracism in their past lives [16]. Second, previous studies primarily used adult sample [9,31]. Abrams et al. [17] found that different age groups exhibited different reactions to cyber-ostracism. Since adolescents have a strong need for social acceptance and affiliation by peers [32], they are more likely to be susceptible and have a stronger negative reaction towards cyber-ostracism [17].

In addition to the inconsistent findings regarding the impact of cyber-ostracism on psychological well-being, prior research only investigated the immediate effect of cyber-ostracism on psychological well-being but did not examine whether cyber-ostracism would have a lasting impact on psychological well-being. Therefore, this study aims to explore the short-term influence of the general situation of cyber-ostracism on adolescents’ psychological well-being. According to the literature, we posit the first hypothesis:

**Hypothesis** **1** **(H1).** *Experience of cyber-ostracism will negatively affect subsequent psychological well-being.*

### 1.2. Rumination as a Mediator

The self-determination theory (SDT) provides a useful framework for understanding the determinants of psychological well-being [33]. SDT posits that fulfilling an individual’s basic psychological needs (i.e., autonomy, competence, and relatedness) is an essential precondition for achieving optimal psychological well-being [34,35]. When these basic psychological needs are threatened, the psychological well-being of individuals will be attenuated [36]. The Temporal Need-Threat Model (TNTM) posits that being ostracized by others will threaten individuals’ four psychological needs (i.e., control, self-esteem, belonging, and meaningful existence) (TNTM; [37]). While the basic psychological needs outlined within the SDT are different from those in TNTM, they overlap in many ways [38]. For example, relatedness describes the need to feel belongingness, intimacy, and connectedness with others, thereby mapping onto the belonging in TNTM [38]. The threatened psychological needs occur in the first stage of the TNTM. Williams named this stage as reflexive stage, during which the responses are immediate and do not vary according to the target’s personality [37]. Although the threatened psychological needs caused by cyber-ostracism are detrimental to psychological well-being, this influence might be weak and temporary [31]. Williams proposed that whether cyber-ostracism can produce lasting effects depends on the extent to which the psychological needs are recovered [37]. The speed of psychological recovery is influenced by the coping strategies adopted to process cyber-ostracism. The process to cope with cyber-ostracism is called the reflective stage in TNTM [37]. When individuals do not effectively apply necessary coping strategies to fortify their threatened psychological needs, they will enter a so-called resignation stage in which their psychological well-being will be attenuated. Rumination has been identified as a maladaptive coping strategy that could hinder psychological recovery following cyber-ostracism [39].

Rumination is defined as the engagement of passive and repeated thinking of distressful events and visualizing their possible causes, consequences, and symptoms [40]. Several reasons can be used to explain why cyber-ostracism elicits ruminative tendencies. Firstly, according to the Stress-Reactive Model of Rumination [41], rumination is a maladaptive coping strategy in response to the experience of stressful events. For example, a three-week longitudinal study found that cyber-victimization was positively associated with rumination [42]. Given that cyber-ostracism is a common stressful experience [16,43], it may lead to rumination. Secondly, the TNTM proposed that ostracism will lead to an increased level of negative affect [37]. Several studies have also confirmed the effect of ostracism on negative affect under a social media context [9,10]. Watkins argued that negative effect was a significant factor that would prompt and intensify ruminative thoughts [44]. Thirdly, according to the Elaborated Control Theory [44], rumination occurs when people recognize that there is a discrepancy between desired goals and current states. Adolescents aim to maintain the feeling of permanent connection through SNSs [9]. However, cyber-ostracism makes adolescents feel isolated, which causes a discrepancy between desired goals (permanent connection) and current states (cyber-ostracism). The attempt to reduce discrepancies will cause an individual’s rumination [44]. Overall, the experience of cyber-ostracism will elicit rumination.

According to the Response Styles Theory [45], rumination continuously directs an individual’s attention to a distressful event with a non-accepting attitude, at the same time preventing any action or behavior that might divert the individual’s attention from this event. Therefore, rumination prevents individuals from recovering from the distressing event and prolongs its adverse effects [46]. For example, a laboratory experiment indicated that ostracized individuals who engaged in ruminative thinking suffered more from threatened needs than those who distracted themselves [47]. In another study, He et al. [48] found that rumination over workplace ostracism experiences would slow psychological recovery and cause low job satisfaction, burnout, and turnover intentions. Therefore, we can conclude that rumination may obstruct the recovery of threatened psychological needs for ostracized adolescents, which in turn impairs their psychological well-being. Hence, we can reasonably formulate the following hypothesis:

**Hypothesis** **2** **(H2).** *Rumination plays a mediating role in the relationship between cyber-ostracism and psychological well-being.*

### 1.3. Mindfulness as Moderator

Despite the fact that cyber-ostracism may impact adolescents’ psychological well-being via rumination, it seems unlikely that all adolescents are equally influenced. The heterogeneity might originate from protective factors such as mindfulness. Mindfulness is an attribute of consciousness that directs individuals’ attention to concentrate and focus on the current moment with a non-judgmental and accepting attitude [49]. Although mindfulness is considered as a psychological trait that most people possess, individuals may vary from one another in their capacity to be mindful [49]. Numerous studies suggested that people who had a high level of mindfulness were less likely to engage in ruminative thinking [50,51,52]. According to the Monitor and Acceptance Theory (MAT), the central mechanisms by which mindfulness works are attention monitoring and acceptance [53]. Attention monitoring brings individuals’ full attention to the current moment rather than dwelling on the past stressful experiences. In fact, mindfulness is sometimes seen as a kind of attention regulation, in that people who practice mindfulness are able to divert their attention from past stresssful events and focus on the present experience [50,53]. Acceptance describes an attitude of openness and non-judgment toward stressful experiences [53]. People who are mindful can notice the stressful events with an acceptive attitude. The acceptive attitude can stop people from lingering on the stressful events and the corresponding symptoms [50,54]. Additionally, individuals with high mindfulness can detect the discrepancy between desired goals and current states, at the same time willing to accept the discrepancy [55], which eventually will reduce the probability and degree of rumination.

Overall, cyber-ostracized individuals possessing a high level of mindfulness are less likely to view the stressful experience and its symptoms as threatening and unacceptable, which reduces the probability of ruminative thinking. Therefore, cyber-ostracized adolescents with high mindfulness can recover from the threatened psychological needs with less difficulty, which in turn inhibits the detrimental impact of cyber-ostracism on their psychological well-being. We could deduce that mindfulness moderates the influence of cyber-ostracism on psychological well-being as well as the correlation between cyber-ostracism and rumination. Furthermore, if rumination mediates the effect of cyber-ostracism on psychological well-being, then the mediating role of rumination may be moderated by mindfulness.

**Hypothesis** **3** **(H3).** *Mindfulness moderates the negative association between cyber-ostracism and psychological well-being, such that individuals with higher mindfulness are less affected by cyber-ostracism than individuals with lower mindfulness.*

**Hypothesis** **4** **(H4).** *Mindfulness moderates the positive association between cyber-ostracism and rumination. Specifically, the positive association between cyber-ostracism and rumination would be weaker among individuals with higher mindfulness.*

**Figure 1:**
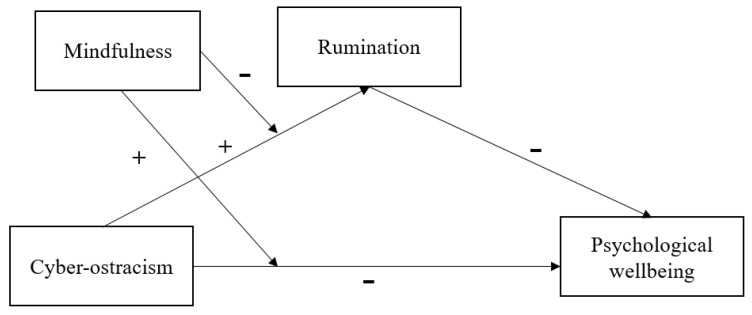
The proposed moderated mediation model.

Overall, the aim of the current research is to investigate the short-term effect of cyber-ostracism on adolescents’ psychological well-being as well as its underlying mechanisms. Specifically, a moderated mediation model (Figure 1) was established to examine (a) whether cyber-ostracism would negatively influence adolescents’ psychological well-being; (b) whether rumination would mediate this influence; and, followed by, (c) whether mindfulness would moderate this influence.

## 2. Participants and Materials

### 2.1. Participants and Procedure

Participants comprised seventh to ninth graders from a middle school in a city in central China. The middle school was chosen according to convenience. Data were collected by distributing a paper-and-pencil questionnaire at school during regular school hours. The students were approached in their classrooms and invited to fill out the questionnaire. Two trained master students helped us to collect the data. Different instruments were administered at each time point to examine the short-term effect of cyber-ostracism on psychological well-being. Cyber-ostracism, rumination, and mindfulness were evaluated at the beginning of the spring semester (Time 1: February 2019). Psychological well-being was assessed three months later (Time 2: May 2019). Specifically, 442 students from grade seven to grade nine participated in Time 1 (48.19% girls; M_age_ = 14.19, SD = 0.94), and 439 students from the same grade participated in Time 2 (48.29% girls; M_age_ = 14.18, SD = 0.97). We matched the two-wave data according to the reported demographic information (i.e., name, gender, age, class, and grade). Fifteen students from the first-wave survey were dropped because they were absent on the day of the administration of the second-wave survey. This procedure yielded a sample of 427 students (48.24% girls; M_age_ = 14.19, SD = 0.95).

A data screening procedure was performed to exclude the invalid sample. In the first-wave survey, an attention check item that reads, “please select 1 if you are a male, 5 if you are a female”, was embedded in the questionnaire to identify careless respondents. Four participants were excluded because they failed the attention check. Two participants without social media accounts (e.g., Weibo, QQ, and WeChat) were also removed. The final sample consists of 421 adolescents (48.22% girls; M_age_ = 14.20, SD = 0.95, range = 12–17 years).

The participants were informed that the data would be used anonymously and all personal information would be kept strictly confidential. The corresponding author’s university granted ethical approval for the survey. Before the paper-and-pencil questionnaire package was completed, we obtained consent from the school, parents, and participants. There were no missing data in this sample.

The relevant data and materials for this study are available on the Open Science Framework (OSF) at the following link: https://osf.io/8rzm7?view_only=8a42c897d1234c37889307bc519c2bf5 (accessed on 17 January 2022).

### 2.2. Measures

**Cyber-ostracism**. Cyber-ostracism was evaluated by the Cyber-ostracism Experience Scale (CES) developed by Niu et al. [15,16]. Respondents were asked to rate how often in the past month they had experienced different forms of cyber-ostracism (e.g., “I get no response when I send notifications in an online group (such as QQ Group, and WeChat group)”). This scale consists of 14 items with a 4-point Likert-type that ranged from 1 (*never*) to 4 (*always*), with higher mean scores reflecting the higher occurrence of cyber-ostracism. The CES has been successfully applied in Chinese adolescents [16]. In the current study, this scale revealed good reliability (Cronbach’s α = 0.895).

**Rumination**. A 10-item Ruminative Response Scale (RRS) was applied to evaluate rumination [56]. The 10-item RRS was extracted from the 22-item RRS [45] and was less contaminated by depressive symptoms (e.g., “Go someplace alone to think about your feelings”). Participants responded on a 4-point Likert scale that ranged from 1 (*never*) to 4 (*always*), with higher average scores reflecting higher occurrence of ruminative thinking. Specifically, the instructions are: “The following items describe people’s reactions after being cyber-ostracized by others. Think back over your experience of cyber-ostracism in the past month and use the rating scale to indicate how well each statement describes your reactions”. The 10-item RRS has good psychometric characteristics in Chinese adolescents [57].

**Mindfulness**. The Child and Adolescent Mindfulness Measure (CAMM) developed by Greco et al. [58] was applied to assess the trait mindfulness (e.g., “It’s hard for me to pay attention to only one thing at a time”). The CAMM consists of 10 items evaluating the thoughts, feelings, and bodily sensations of participants using a 5-point Likert scale (0 = *Never*, 4 = *Always*). The CAMM features good psychometric characteristics in Chinese adolescents [59].

**Psychological well-being**. An 8-item Flourishing Scale (FS) was used to measure adolescents’ psychological well-being (e.g., “I am optimistic about my future”) [60]. Participants reported on a Likert scale that ranged from 1 (*strongly disagree*) to 7 (*strongly agree*). The mean of the items was calculated to obtain a general level of psychological well-being. A previous study showed that the FS possessed good psychometric characteristics in Chinese adolescents [61].

## 3. Results

### 3.1. Analytical Strategy

First, we calculated descriptive statistics and Peterson’s correlation coefficients for all variables using SPSS 22.0. Gender was positively related to mindfulness, and age was negatively associated with rumination. Therefore, gender and age were entered as control variables in the following analyses. Subsequently, we examined the predicted effect of cyber-ostracism on the psychological well-being of adolescents, and tested the mediating role of rumination using Mackinnon’s [62] four-step procedure. Finally, we used the PROCESS macro (version 3.5) to test the moderated mediation model [63].

### 3.2. Preliminary Analyses

Table 1 demonstrates the mean, standard deviation, and Pearson’s correlation coefficients for all variables. Overall, the cyber-ostracism experience of adolescents is relatively low, and adolescents have a relatively high level of psychological well-being. We also report the reliability of the four study variables. As shown in Table 1, all measures have good internal consistency reliability, with Cronbach’s alpha lying well above the suggested threshold of 0.70. In terms of the convergent validity, the composite reliability (CR) of the four measures is above the recommended level of 0.60. However, the average variance extracted (AVE) of cyber-ostracism, rumination, and mindfulness is below the recommended level of 0.50. Fornell and Larcker [64] pointed out that the AVE might be a more conservative estimate of the validity of the measures. They suggested that the convergent is adequate if the CR of the measures meet the suggested level. For example, in Lam’s study [65], CR ranges from 0.71–0.74 and AVE is above 0.31. Putting these pieces together, our measures are reliable.

In the correlation matrix, the cyber-ostracism showed a positive and significant correlation with rumination, and a negative and significant correlation with mindfulness and psychological well-being. Rumination exhibited a negative and significant correlation with mindfulness and psychological well-being. Mindfulness was positively and significantly linked to psychological well-being. The negative relationship between cyber-ostracism and psychological well-being supported Hypothesis 1. The regression analysis also showed that cyber-ostracism at time 1 could significantly predict psychological well-being at time 2 (see Model 1 in Table 2), which further supported Hypothesis 1.

### 3.3. Testing for Mediation Effect

To test the mediation effect of rumination in the relationship between cyber-ostracism and psychological well-being, we adopted a four-step procedure proposed by Mackinnon [62], which needs (a) a significant association between cyber-ostracism and psychological well-being; (b) a significant association between cyber-ostracism and rumination; (c) a significant association between rumination and psychological well-being when controlling for cyber-ostracism; and (d) a significant coefficient for the indirect path between cyber-ostracism and psychological well-being via rumination. In the first three steps, we used the ordinary least squares regression to estimate parameters. The bias-corrected percentile bootstrap (5000 bootstrapped resamples) is used to determine whether the last procedure is satisfied.

Table 2 presents the standardized regression results of the estimated models. The findings indicated that cyber-ostracism was negatively and significantly associated with psychological well-being. Cyber-ostracism was positively and significantly correlated with rumination. When controlling for cyber-ostracism, rumination was still negatively and significantly correlated with psychological well-being. The results of bias-corrected percentile bootstrap revealed that the indirect effect of cyber-ostracism on psychological well-being through rumination was significant (ab = −0.08, SE = 0.02, 95% CI = [−0.12, −0.04]). The mediation effect accounted for 30.77% of the total effect. The results satisfied the criteria for building up the mediation effect. Accordingly, Hypothesis 2 was supported.

### 3.4. Testing for Moderated Mediation

**Figure 2:**
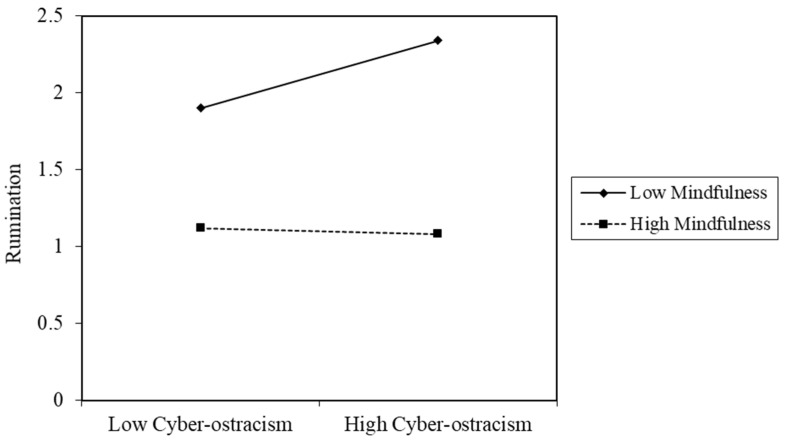
Mindfulness moderated the association between cyber-ostracism and rumination.

**Figure 3:**
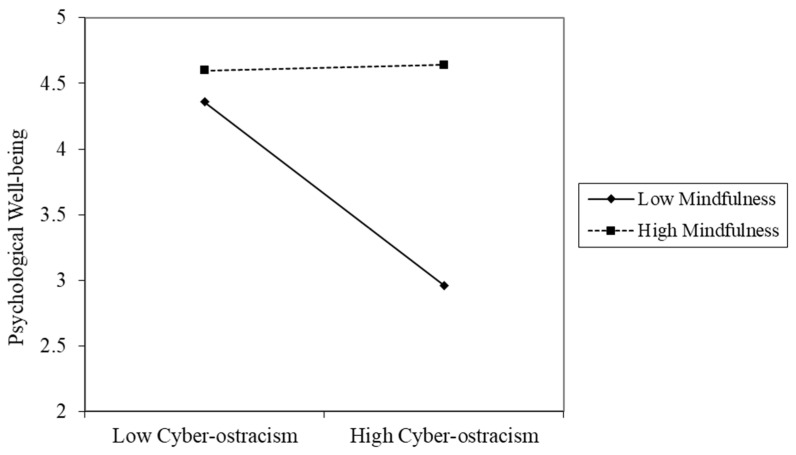
Mindfulness moderated the association between cyber-ostracism and psychological well-being.

We used the PROCESS macro (Model 8) developed by Hayes [63] to examine the moderated effect of mindfulness. The mediator variable model (Model 1) examined the moderation effect of mindfulness on the association between cyber-ostracism and rumination, and the dependent variable model (Model 2) examined the moderated role of mindfulness on the association between cyber-ostracism and psychological well-being. Within both models, gender and age were used as control variables. As shown in Table 3, Model 1 revealed that cyber-ostracism was significantly and positively associated with rumination and the interaction term between cyber-ostracism and mindfulness, which indicated that this association between cyber-ostracism and rumination was moderated by mindfulness (ΔR^2^ = 0.01). For descriptive purpose, we plotted the relationship between rumination and cyber-ostracism, respectively, for both the low and high levels of mindfulness (see Figure 2). Model 2 showed a significant and negative impact of cyber-ostracism on psychological well-being, and this impact was moderated by mindfulness (ΔR^2^ = 0.02). For descriptive purpose, we plotted predicted psychological well-being against cyber-ostracism, respectively for both low and high levels of mindfulness (see Figure 3). These findings exhibited that both the link between cyber-ostracism and rumination and the link between cyber-ostracism and psychological well-being were moderated by mindfulness.

Table 3 showed the conditional direct effect and conditional indirect effect. As for the conditional direct effect, the findings indicated that for low-mindful individuals (1 SD below the mean), higher cyber-ostracism was correlated with lower psychological well-being. However, for high-mindful individuals (1 SD above the mean), the correlation between cyber-ostracism and psychological well-being was non-significant. As for the conditional indirect effect, the results showed that for low-mindful individuals, cyber-ostracism was significantly and negatively related to rumination. However, for high-mindful individuals, the association between cyber-ostracism and rumination was non-significant. Overall, Hypothesis 3 was supported.

## 4. Discussion

This study explored the short-term influence of cyber-ostracism on the psychological well-being of adolescents, and examined its underlying mechanisms by employing a moderated mediation model. The findings revealed a negative effect of cyber-ostracism on the psychological well-being of adolescents. Rumination partially mediated cyber-ostracism’s negative effect on the psychological well-being of adolescents, and mindfulness moderated this negative effect. In addition, mindfulness moderated the mediating effect of rumination. In particular, the indirect effect of cyber-ostracism on the psychological well-being mediated by rumination was significant only for adolescents possessing low and medium levels of mindfulness; this effect was not evident for adolescents with a high level of mindfulness.

First, consistent with Hypothesis 1, the results indicated that cyber-ostracism had a detrimental effect on adolescents’ psychological well-being. To our knowledge, most of the previous research focused on emotional well-being. Only a few studies focused on the impact of cyber-ostracism on psychological well-being [9,31], and they investigated the immediate effect of cyber-ostracism on psychological well-being. The present study investigated the short-term effect of cyber-ostracism and found a similarly weak effect over three months to that reported by Wang et al. [31]; somewhat lower levels of psychological well-being were evident for adolescents reporting more cyber-ostracism three months prior. Although the short-term effect of cyber-ostracism on psychological well-being was weak, it suggested that there were still some adolescents who were unable to recover from the negative experiences of cyber-ostracism. Therefore, it is necessary to investigate the underlying mechanisms of the relationship between cyber-ostracism and psychological well-being, which can help us understand how cyber-ostracism affects psychological well-being and why some adolescents are less affected.

Second, consistent with Hypothesis 2, our results supported the mediating effect of rumination in the negative influence of cyber-ostracism on the psychological well-being of adolescents. This finding is consistent with preceding research indicating that rumination impairs psychological adjustment and hinders recovery from ostracism [47]. Rumination continuously directs an individual’s attention to the cyber-ostracism experiences with a non-accepting attitude, and obstructs the individual from engaging in mood-enhancing activities [39]. Recalling an episode of cyber-ostracism elicits damages similar to experiencing cyber-ostracism [66]. Repetitive thoughts on cyber-ostracism prompt the individual to constantly relive in the anguish caused by cyber-ostracism [67]. Therefore, the ruminative process not only delays the recovery of threatened psychological needs, but also further deteriorates psychological needs. The impaired psychological needs will attenuate psychological well-being. Taken together, our study exposed that cyber-ostracism was associated with rumination, which in turn reduced the psychological well-being of adolescents.

Third, consistent with Hypothesis 3, our study confirmed the moderating effect of mindfulness on the relationship between cyber-ostracism and adolescents’ psychological well-being. Specifically, for individuals possessing a high level of mindfulness, the effect of cyber-ostracism on psychological well-being was shown to be non-significant. One explanation is that mindfulness can direct adolescents’ attention to present-moment experience and accelerate the recovery of basic psychological needs that were threatened by ostracism [67].

Fourth, consistent with Hypothesis 4, the results revealed that mindfulness played a moderating role in the relationship between cyber-ostracism and rumination. That is, the relationship between cyber-ostracism and rumination was contingent upon adolescents’ level of mindfulness, such that this relationship was non-significant for young individuals possessing a high level of mindfulness. Mindfulness can direct adolescents’ attention towards the present-moment experience with a non-judgmental and accepting attitude, which in turn can help adolescents break their habitual ruminative cycle and shift their attention away from the discrepancies [46]. Therefore, adolescents with a high level of mindfulness can better cope with cyber-ostracism and its detrimental effects.

## 5. Limitations

We acknowledge that several limitations are inherent in this research. First, participants in the present study all attended the same middle school, which limits the overall representativeness of the population. Future research should recruit adolescents from different cultural backgrounds and different establishments to enhance the representativeness of the population. Second, all instruments in the current study were self-reported, which may lead to social desirability and common method biases. Future research should use various instruments such as SNSs recorded data and third-party observation to reduce biases. Third, the present study hypothesized that rumination hindered the recovery of threatened psychological needs caused by cyber-ostracism, so that the psychological well-being of adolescents was impaired. However, the mediating role of psychological needs was not examined in this study. Future research can consider investigating the role of psychological needs in the process of impaired psychological well-being caused by cyber-ostracism. Fourth, although the study variables have established significant links, the strength of the associations is relatively weak. For instance, the correlation between cyber-ostracism and rumination is only 0.22. One possible explanation is that we asked adolescents to report their rumination for the cyber-ostracism experiences of the past month. For the cyber-ostracism experiences that occurred earlier, adolescents might not recall the psychological state at the time. Future research can investigate the experiences of cyber-ostracism in the last week and examine its effects on rumination and psychological well-being. Fifth, we used the sequential design to evaluate the short-term effect of cyber-ostracism for adolescents and examine the underlying mechanisms. Although the sequential design has an advantage in common with the longitudinal design in allowing time to pass, it does not take into account the previous levels of the variables and, therefore, does not account for autoregressive effects [68]. Based on the sequential design, we may not draw rigorous causal inferences. Therefore, we should be cautious in explicating our findings, as we may not be able to rule out alternative causal effects. In order to establish causality, longitudinal designs that measure all key variables simultaneously at each time point are needed to further test our theoretical model. Finally, when examining the association between cyber-ostracism and psychological well-being, we did not control for in-person ostracism. Although SNSs have gradually become a main social tool for adolescents, we should not ignore the ostracism caused by face-to-face communication. Future research could control for in-person ostracism, to more accurately estimate the effect of adolescents’ cyber-ostracism on their psychological well-being.

## 6. Conclusions

This study has made numerous theoretical and practical contributions. As for theoretical contributions, the present study revealed the short-term effect of the general situation of cyber-ostracism on the psychological well-being of adolescents. In addition, the current study uncovered the underlying mechanisms between cyber-ostracism and adolescents’ psychological well-being by investigating the mediating effect of rumination and the moderator moderating role of mindfulness. This research will help scholars understand how and when cyber-ostracism impairs psychological well-being in adolescents. From a practical perspective, adolescents should be aware of the detrimental impact of cyber-ostracism on psychological well-being as well as reduce the exclusion and marginalization of others. As for adolescents who experience cyber-ostracism, parents and educators should give them guidance and support to reduce their ruminative thinking [69]. Given that mindfulness can both buffer the direct link between cyber-ostracism and psychological well-being and the indirect link between cyber-ostracism and psychological well-being via the mediation of rumination, mindfulness-based interventions should be adopted to promote the personal trait of mindfulness in adolescents.

## Figures and Tables

**Table 1:** Descriptive statistics and Pearson’s correlation coefficients.

Variables	M	SD	α	CR	AVE	1	2	3	4	5
1. Gender	0.48	0.50	-	-	-					
2. Age	14.20	0.95	-	-	-	−0.08				
3. Cyber-ostracism	1.89	0.58	0.90	0.90	0.39	−0.06	0.06			
4. Rumination	2.41	0.61	0.86	0.86	0.39	0.09	0.12 *	0.22 ***		
5. Mindfulness	2.29	0.75	0.82	0.83	0.33	−0.16 **	−0.05	−0.20 ***	−0.66 ***	
6. Psychological well-being	4.80	1.19	0.91	0.91	0.56	0.03	0.03	−0.26 ***	−0.39 ***	0.43 ***

Note. N = 421. Gender was coded as binary variable (0 = boy and 1 = girl). α = Cronbach’s alpha; CR = composite reliability; AVE = average variance extracted. * *p* < 0.05, ** *p* < 0.01, *** *p* < 0.001.

**Table 2:** Examining the mediation effect of cyber-ostracism on psychological well-being.

Predictors	Model 1 (PS)			Model 2 (Rumination)			Model 3 (PS)		
	*β*	*t*	*p*	*β*	*t*	*p*	*β*	*t*	*p*
Gender	0.02	0.32	0.75	0.11	2.42	0.02	0.06	1.23	0.21
Age	0.04	0.92	0.36	0.12	2.42	0.02	0.09	1.91	0.06
Cyber-ostracism	−0.26	−5.45	0.00	0.22	4.69	0.00	−0.18	−3.90	0.00
Rumination							−0.36	−7.90	0.00
R^2^	0.06			0.07			0.18		
F	10.15		0.00	10.04		0.00	24.34		0.00

Note. N = 421. PS = Psychological well-being.

**Table 3:** Examining the moderated mediation effect of cyber-ostracism on psychological well-being.

	*β*	*SE*	*t*	*p*
Model 1: Mediator variable model				
Gender	0.01	0.05	0.11	0.91
Age	0.06	0.02	2.38	0.18
Cyber-ostracism	0.10	0.04	2.49	0.01
Mindfulness	−0.51	0.03	−16.58	0.00
Cyber-ostracism × Mindfulness	−0.12	0.04	−2.81	0.01
R^2^ = 0.46, F = 71.14 (*p* = 0.00)				
Model 2: Dependent variable model				
Gender	0.20	0.10	1.92	0.05
Age	0.09	0.05	1.67	0.09
Cyber-ostracism	−0.34	0.09	−3.82	0.00
Rumination	−0.27	0.11	−2.50	0.01
Mindfulness	0.48	0.09	5.28	0.00
Cyber-ostracism × Mindfulness	0.36	0.10	3.52	0.00
R^2^ = 0.28, F = 26.36 (*p* = 0.00)				
Conditional direct effect analysis at Mindfulness = M ± SD	*β*	*Boot SE*	*BootLLCI*	*BootULCI*
M − 1 SD (−0.75)	−0.61	0.12	−0.84	−0.37
M − 1 SD (0.00)	−0.34	0.09	−0.51	−0.16
M + 1 SD (0.75)	−0.07	0.11	−0.29	0.15
Conditional indirect effect analysis at Mindfulness = M ± SD	*β*	*Boot SE*	*BootLLCI*	*BootULCI*
M − 1 SD (−0.75)	−0.05	0.03	−0.11	−0.08
M − 1 SD (0.00)	−0.03	0.02	−0.06	−0.002
M + 1 SD (0.75)	−0.001	0.01	−0.03	0.03

Note. N = 421. LL = low limit, UL = upper limit, CI = confidence interval.

## Data Availability

The data in this study are available on request from the corresponding author and are not publicly accessible due to privacy concerns.
